# Cytokine secretion in breast cancer cells – MILLIPLEX assay data

**DOI:** 10.1016/j.dib.2019.104798

**Published:** 2019-11-14

**Authors:** Karin Chen, Leo Satlof, Guillaume Stoffels, Udithi Kothapalli, Noah Ziluck, Maribel Lema, Leonid Poretsky, Dimiter Avtanski

**Affiliations:** a Friedman Diabetes Institute at Lenox Hill Hospital, Northwell Health, New York, NY, USA; b The Feinstein Institutes for Medical Research, Manhasset, NY, USA; c Donald and Barbara Zucker School of Medicine at Hofstra/Northwell, Hempstead, NY, USA

**Keywords:** Breast cancer, Cytokine, Inflammation, Tumor microenvironment

## Abstract

Metastatic breast cancer is the most advanced stage of breast cancer and the leading cause of breast cancer mortality. Although understanding of the cancer progression and metastasis process has improved, the bi-directional communication between the tumor cell and the tumor microenvironment is still not well understood. Breast cancer cells are highly secretory, and their secretory activity is modulated by a variety of inflammatory stimuli present in the tumor microenvironment. Here, we characterized the cytokine expression in human breast cancer cells (MDA-MB-231, MCF-7, T-47D, and BT-474) *in vitro* using 41 cytokine MILLIPLEX assay. Further, we compared cytokine expression in breast cancer cells to those in non-tumorigenic human breast epithelial MCF-10A cells.

**Table:** Specifications Table

Subject area	*Biology*
More specific subject area	Oncology
Type of data	Tables
How data was acquired	MILLIPLEX assay
Data format	Raw and analyzed
Experimental factors	Cytokine levels in conditioned medium of human breast cancer and non-tumorigenic breast epithelial cells harvested at 24 and 48 hours.
Experimental features	Cytokine levels in cell culture conditioned medium from various human breast cancer cell lines (MDA-MB-231, MCF-7, T-47D, and BT-474 cells) were measured and compared to those from non-tumorigenic breast epithelial MCF-10A cells.
Data source location	New York, New York, USA
**Data accessibility**	On a public repository Repository name: Mendeley Data Data identification number: https://doi.org/10.17632/tvt8zm37w5.2 Direct URL to data: https://doi.org/10.17632/tvt8zm37w5.2

**Table:** 

**Value of the Data** • Breast cancer cells are highly secretory cells producing variety of cytokines and other molecules that can modulate or are modulated by the tumor microenvironment. • Inflammation mediates the initiation and progression of breast cancer, including directly affecting secretory activity of the cancer cells. • Understanding the bi-directional communication between tumor microenvironment and the cancer cells can lead to the development of novel therapies for breast cancer.

## 1 Data

### 1.1 Basal cytokine secretion in human breast cancer and non-tumorigenic breast epithelial cells

To evaluate basal cytokine secretion in breast cancer cells, we selected four different human breast cancer cell lines (MDA-MB-231, MCF-7, T-47D, and BT-474) possessing various molecular characteristics and metastatic potential. We also selected non-tumorigenic human breast epithelial MCF-10A cell line for comparison. The characteristics of each cell line used are listed in Table 1. We maintained the cells in serum-free medium for 24 and 48 hours and measured the levels of 41 cytokines (listed in Table 2) secreted in the medium using MILLIPLEX assay. The data from the assay are listed in Table 3. Further, we compared the level of each cytokine secreted from each breast cancer cell line relative to the secretion from MCF-10A cells (Table 4).

**Table 1:** Categorization and molecular characteristics of the cell lines used.

Cell Line	ER	PR	HER2	BRCA1	Subtype	Tumor
MCF-10A	–	–	–	WT	B	N/A
MDA-MB-231	–	–	–	WT	TNB	AC
MCF-7	+	+	–	WT	LA	IDC
T-47D	+	+	–	WT	LA	IDC
BT-474	+	+	+	WT	LB	IDC

[1,2] ER, estrogen receptor; PR, progesterone receptor; HER2, human epidermal growth factor receptor 2; BRCA1, breast cancer gene 1; WT, wild type; TNB, triple-negative B; B, basal; LA, luminal A; LB, luminal B; AC, adenocarcinoma; IDC, invasive lobular carcinoma.

**Table 2:** List of cytokines measured by MILLIPLEX assay.

Abbreviation	Full name	Synonyms
TNFα	Tumor Necrosis Factor Alpha	TNF-α, TNF-alpha, TNFA, TNF, TNFSF2, TNLG1F, DIF
TNFβ	Tumor Necrosis Factor Beta	TNF-β, TNF-beta, TNFB, TNFSF1, LT, TNLG1E, Lymphotoxin-α, Lymphotoxin Alpha, LTA
IFN-α2	Interferon Alpha 2	IFNA, IFNA2, IFNA2B, IFN-alphaA, INDA2
IFN-γ	Interferon gamma	IFNγ, IFNgamma, IFN-gamma, IFN-g, IFI, TCRalpha
CCL2	Chemokine (C–C Motif) Ligand 2	MCP-1, MCP1, GDCF-2, HC11, HSMCR30, MCAF, SCYA2, SMC-CF
CCL3	Chemokine (C–C Motif) Ligand 3	MIP-1α, MIP-1-alpha, MIP1A, SCYA3, G0S19-1, LD78ALPHA
CCL4	Chemokine (C–C Motif) Ligand 4	MIP-1β. MIP-1-beta, MIP1B, MIP1B1, SCYA2, SCYA4, ACT2, AT744.1, G-26, HC21, LAG-1, LAG1
CCL5	Chemokine (C–C Motif) Ligand 5	RANTES, eoCP, SCYA5, TCP228, D17S136E, SIS-delta, SISd
CCL7	Chemokine (C–C Motif) Ligand 7	CCL7, MCP-3, MCP3, FIC, MARC, NC28, SCYA6, SCYA7
CCL11	C–C Motif Chemokine Ligand 2	Eotaxin-1, HC11, MCAF, MCP1, MCP-1, SCYA2, GDCF-2, SMC-CF, HSMCR30
CCL22	C–C Motif Chemokine 22	MDC, ABCD-1, SCYA22, STCP-1, DC/B-CK, A-152E5.1
CXCL1	C-X-C Motif Chemokine Ligand 1	Groucho, GRO, GRO1, GROA, GROa, MGSA, MGSA-alpha, MGSA-A, MGSA-a, NAP-3, SCYB1, FSP
CXCL10	C-X-C Motif Chemokine Ligand 10	IP-10, INP10, IFI10, SCYB10, gIP-10, C7, crg-2, mob-1
CX3CL1	Chemokine (C-X3-C Motif) Ligand 1	Fractalkine, Neurotactin, ABCD-3, C3Xkine, CXC3, CXC3C, NTN, NTT, SCYD1
IL-1α	Interleukin 1 Alpha	IL1α, IL-1alpha, IL1alpha, IL1-ALPHA, IL-1A, IL1A, IL1, Hematopoietin 1
IL-1β	Interleukin 1 Beta	IL1-beta, IL1beta, IL1-BETA, IL-1, IL1, IL1F2
IL-2	Interleukin 2	IL2, TCGF, Lymphokine
IL-3	Interleukin 3	IL3, MCGF, MULTI-CSF
IL-4	Interleukin 4	IL4, BCGF-1, BCGF1, BSF-1, BSF1
IL-5	Interleukin 5	IL5, EDF, TRF
IL-6	Interleukin 6	IL6, CDF, HGF, HSF, BSF-2, BSF2, IFNB2, IFN-beta-2
IL-7	Interleukin 7	IL7
IL-8	Interleukin 8	IL8, CXCL8, NAF, GCP-1, GCP1, LECT, LUCT, NAP-1, NAP1, GCP-1, LYNAP, MDNCF, MONAP
IL-9	Interleukin 9	IL9, HP40, P40
IL-10	Interleukin 10	IL10, IL10A, CSIF, GVHDS, TGIF
IL-12p40	Interleukin 12 Subunit p40	IL12B, IL-12B, CLMF, CLMF2, IMD28, IMD29, NKSF, NKSF2
IL-12p70	Interleukin 12 p70 (active heterodimer)	
IL-13	Interleukin 13	IL13, P600
IL-15	Interleukin 15	IL15
IL-17A	Interleukin 17A	IL17A, IL-17, IL17, CTLA-8, CTLA8
IL-1RA	Interleukin 1 Receptor Antagonist	IL1RA, IL-1ra, IL-1ra3, IL1RN, IL-1RN, IL1F3, ICIL-1RA, MVCD4, DIRA, IRAP
G-CSF	Granulocyte Colony-Stimulating Factor	GCSF, CSF3, CSF3OS, C17orf33
GM-CSF	Granulocyte-Macrophage Colony-Stimulating Factor	CSF2, CSF
FLT3L	FMS-Like Tyrosine Kinase 3 Ligand	FL, FLG3L, FLT3LG
sCD40L	Soluble Cluster of Differentiation 40 Ligand	
EGF	Epidermal Growth Factor	HOMG4, URG
FGF-2	Fibroblast Growth Factor 2	FGF2, FGFB, BFGF, HBGF-2
TGFα	Transforming Growth Factor Alpha	TGFA
PDGF-AA	Platelet-Derived Growth Factor AA Homodimer	
PDGF-AB/BB	Platelet-Derived Growth Factor AB/BB Homodimers	
VEGF-A	Vascular Endothelial Growth Factor A	VEGFA, VEGF, VPF, MVCD1

**Table 3:** Basal cytokine secretion in breast cancer cell lines.

Cytokine	24h					48h				
	MCF-10A	MDA-MB-231	MCF-7	T-47D	BT-474	MCF-10A	MDA-MB-231	MCF-7	T-47D	BT-474
TNFα	<2.950	7.275 (±0.179)	<2.560	45.895 (±11.591)	15.328 (±0.434)	4.103 (±0.109)	14.935 (±0.546)	<2.560	33.630 (±9.101)	13.898 (±2.355)
TNFβ	<2.485	<2.493	<2.260	<2.348	<2.260	<2.735	<3.620	<2.260	<2.398	<2.398
IFN-α2	<5.088	<13.510	<4.845	<7.805	<8.313	<7.425	<13.278	<4.970	12.123 (±3.010)	14.998 (±2.485)
IFN-γ	3.843 (±0.414)	8.290 (±0.655)	<1.860	4.948 (±0.463)	<1.875	3.328 (±0.292)	7.790 (±0.533)	<1.860	5.848 (±1.211)	<3.865
CCL2	829.865 (±58.989)	1069.333 (±47.394)	<3.020	3337.000 (±373.176)	270.170 (±4.571)	1665.750 (±100.659)	2197.500 (±90.143)	<3.228	2739.750 (±683.225)	616.520 (±146.576)
CCL3	<7.930	10.745 (±0.345)	<7.930	<7.930	<7.930	<7.930	21.803 (±0.770)	<7.930	<7.930	<7.930
CCL4	4.370 (±0.167)	7.793 (±0.231)	<2.890	43.030 (±8.784)	3.985 (±0.170)	5.028 (±0.153)	12.783 (±0.106)	<2.890	26.075 (±5.572)	4.735 (±0.747)
CCL5	8.970 (±0.143)	102.598 (±1.836)	5.545 (±0.236)	232.223 (±19.932)	154.808 (±1.343)	15.265 (±0.452)	180.860 (±5.135)	6.910 (±0.678)	226.218 (±38.228)	370.975 (±81.655)
CCL7	12.333 (±0.661)	12.373 (±0.856)	<3.188	10.858 (±1.531)	4.708 (±0.336)	13.038 (±0.279)	14.145 (±1.080)	<3.188	8.675 (±1.097)	6.263 (±1.233)
CCL11	9.215 (±0.193)	9.470 (±0.254)	<2.990	3.845 (±0.245)	4.183 (±0.353)	9.515 (±0.325)	10.098 (±0.232)	<2.990	<3.915	4.118 (±0.210)
CCL22	9.205 (±1.055)	12.430 (±0.430)	8.713 (±0.541)	12.753 (±1.462)	17.815 (±1.015)	10.213 (±0.642)	14.900 (±1.402)	10.345 (±0.179)	10.750 (±1.686)	68.720 (±21.248)
CXCL1	8732.750 (±605.110)	4522.000 (±156.844)	4.068 (±0.408)	456.633 (±121.228)	387.795 (±9.656)	7983.750 (±551.861)	10,511.000 (±762.600)	<3.688	249.928 (±70.653)	1451.665 (±413.137)
CXCL10	143.163 (±5.162)	372.870 (±16.491)	<1.655	2964.750 (±991.557)	75.195 (±2.178)	151.400 (±5.605)	778.045 (±52.223)	5.035 (±0.430)	2951.205 (±840.243)	207.890 (±63.633)
CX3CL1	49.290 (±3.842)	164.840 (±4.438)	21.543 (±1.511)	44.715 (±2.424)	92.060 (±6.627)	60.225 (±2.750)	323.065 (±11.234)	20.505 (±0.913)	52.313 (±8.922)	336.315 (±116.011)
IL-1α	8.985 (±0.297)	17.460 (±0.187)	<2.560	5.265 (±0.220)	<2.560	12.563 (±1.887)	35.945 (±2.826)	<2.560	6.508 (±0.554)	<2.560
IL-1β	<2.770	<2.770	<2.770	<2.770	<2.770	<2.770	<2.770	<2.770	<2.770	<2.770
IL-2	<2.670	<2.670	<2.670	<2.670	<2.670	<2.670	<2.670	<2.670	<2.670	<2.670
IL-3	<2.570	<2.570	<2.570	<2.570	<2.570	<2.570	<2.570	<2.570	<2.570	<2.570
IL-4	74.775 (±6.307)	61.715 (±6.151)	<2.703	14.198 (±2.139)	8.208 (±0.502)	77.108 (±6.463)	87.700 (±4.876)	<2.703 (±0.415)	9.453 (±2.059)	22.758 (±6.739)
IL-5	<2.790	<2.790	<2.790	<2.790	<2.790	<2.790	<2.790	<2.790	<2.790	<2.790
IL-6	21.295 (±0.856)	4583.250 (±119.634)	<2.280	<2.280	3.843 (±0.266)	46.883 (±4.309)	5391.00 (±502.887)	<2.280	<2.485	4.423 (±0.194)
IL-7	4.725 (±0.540)	16.398 (±1.352)	2.728 (±0.759)	5.920 (±1.182)	7.343 (±1.935)	6.728 (±0.849)	17.173 (±1.438)	<1.820	6.668 (±1.515)	6.578 (±1.174)
IL-8	6048.000 (±438.163)	5568.250 (±291.235)	<1.750	24.558 (±8.036)	278.205 (±11.059)	>6805.00	00	<1.750	33.570 (±8.789)	454.288 (±99.778)
IL-9	<2.980	<2.980	<2.980	<3.595	<2.980	<2.980	<2.980	<2.980	5.158 (±0.626)	<2.980
IL-10	2.708 (±0.226)	9.503 (±0.792)	<1.080	2.648 (±0.407)	<1.473	3.005 (±0.279)	12.180 (±1.267)	<1.080	2.825 (±0.354)	<2.133
IL-12p40	4.780 (±0.302)	6.583 (±0.465)	<2.415	6.785 (±0.546)	3.388 (±0.269)	5.543 (±0.298)	7.803 (±0.252)	<1.868	5.883 (±0.604)	4.188 (±0.393)
IL-12p70	<2.083	3.228 (±0.263)	<1.300	<1.640	<1.355	<2.293	4.128 (±0.333)	<1.193	<2.020	<1.793
IL-13	3.253 (±0.198)	5.945 (±0.302)	<1.945	6.325 (±0.243)	<2.235	5.628 (±0.276)	7.975 (±0.220)	<1.940	5.893 (±0.912)	<3.675
IL-15	4.103 (±0.278)	15.948 (±0.280)	<0.820	4.493 (±0.870)	1.845 (±0.054)	6.758 (±0.192)	25.093 (±0.447)	<0.908	3.803 (±0.633)	3.483 (±0.503)
IL-17A	1.963 (±0.184)	2.670 (±0.192)	<1.340	<1.743	<1.418	2.130 (±0.178)	3.145 (±0.485)	<1.340	<1.798	<1.645
IL-1RA	8.918 (±0.528)	18.138 (±0.941)	4.168 (±0.477)	9.645 (±1.276)	5.593 (±0.361)	13.190 (±1.039)	24.433 (±1.700)	4.093 (±0.299)	9.313 (±1.333)	14.175 (±4.190)
G-CSF	357.355 (±31.119)	337.920 (±6.059)	4.438 (±0.224)	52.563 (±4.055)	30.685 (±5.760)	428.770 (±42.610)	1464.250 (±181.843)	5.638 (±0.299)	74.585 (±11.091)	34.760 (±7.455)
GM-CSF	29.400 (±1.513)	1070.000 (±16.678)	<2.920	7.968 (±0.800	<2.978	37.430 (±3.884)	3871.750 (±101.647)	<2.920	8.048 (±1.245)	<4.253
FLT3L	8.170 (±0.318)	16.123 (±0.289)	<2.915	11.138 (±1.771)	8.370 (±0.320)	9.973 (±0.365)	24.038 (±0.268)	<2.798	7.940 (±1.345)	19.983 (±3.394)
sCD40L	3.503 (±0.580)	5.683 (±0.739)	<2.010	4.418 (±0.644)	<2.078	4.225 (±0.379)	7.445 (±0.859)	<2.010	4.448 (±1.000)	<3.158
EGF	5.685 (±0.172)	6.845 (±0.296)	3.853 (±0.219)	4.637 (±0.257)	4.883 (±0.206)	6.053 (±0.160)	7.095 (±0.409)	3.725 (±0.134)	5.038 (±0.578)	<4.068
FGF-2	31.070 (±3.084)	46.325 (±3.386)	9.380 (±1.099)	28.823 (±3.851)	15.995 (±1.279)	161.310 (±3.588)	42.680 (±3.098)	10.745 (±1.721)	25.620 (±4.764)	25.545 (±4.515)
TGFα	<2.800	10.168 (±0.210)	9.348 (±0.247)	8.935 (±1.131)	<2.800	<2.800	13.255 (±0.373)	14.285 (±0.249)	6.033 (±0.627)	3.798 (±0.445)
PDGF-AA	55.013 (±2.143)	85.943 (±1.684)	42.038 (±0.879)	61.323 (±17.375)	45.478 (±0.624)	99.003 (±3.448)	179.295 (±1.338)	104.150 (±3.264)	42.478 (±11.780)	665.713 (±217.050)
PDGF-AB/BB	12.120 (±0.833)	37.260 (±0.721)	201.665 (±13.144)	417.443 (±163.571)	28.715 (±0.964)	12.195 (±0.797)	74.628 (±1.039)	617.865 (±6.517)	270.130 (±67.436)	1296.283 (±434.299)
VEGF-A	151.368 (±5.500)	1775.415 (±112.696)	98.468 (±3.220)	265.685 (±76.573)	216.440 (±3.643)	276.750 (±9.789)	2070.230 (±51.826)	219.028 (±5.175)	248.513 (±50.523)	316.485 (±8.822)

Cytokine levels [pg/ml] in cell culture medium collected after 24 and 48 hours, measured by MILLIPLEX assay. Each value represents the mean of measurements made in 4 setups. Some setup measurements were below or above assay detection level. If one or more of the 4 measurements in a group were below (above) detection level, then those values were assigned the threshold level and the corresponding mean was reported using the “less than” (“greater than”) symbol. Note that there was no situation in which the 4 setup measurements contained values both above and below detection level.

**Table 4:** Comparison of cytokine expression between the four breast cancer cell lines and breast non-tumorigenic epithelial cells.

Cytokine	24h						48h					
	MCF-10A	MDA-MB-231	MCF-7	T-47D	BT-474	Statistics	MCF-10A	MDA-MB-231	MCF-7	T-47D	BT-474	Statistics
TNFα	<1.548	2.862 (±0.036)	<1.356	5.418 (±0.315)	3.936 (±0.040)	a, p = 0.0012, ** b, p < 0.0001, **** d, p < 0.0001, **** e, p < 0.0001, **** f, p < 0.0001, **** g, p 0.0002, *** h, p < 0.0001, **** i, p < 0.0001, **** j, p < 0.0001, **** k, p = 0.0034, **	2.035 (±0.039)	3.898 (±0.053)	<1.356	4.893 (±0.422)	3.732 (±0.252)	a, p = 0.0022, ** b, p < 0.0001, **** c, p < 0.0001, **** d, p = 0.0005, *** e, p = 0.0006, *** f, p < 0.0001, **** g, p = 0.0578, NS h, p = 0.5437, NS i, p < 0.0010, *** j, p < 0.0006, *** k, p = 0.0561, NS
TNFβ	<1.307	<1.516	<1.176	1.228	<1.176	a, p = 0.2416, NS b, p = 0.0503, NS d, p = 0.6667, NS g, p = 0.1852, NS	<1.429	<1.802	<1.176	<1.255	<1.255	a, p = 0.1000, NS
IFN-α2	<2.141	<2.977	<2.201	<2.511	<2.536	a, p = 0.9485, NS	<2.486	<2.968	<2.117	3.564 (±0.180)	3.776 (±0.342)	a, p = 0.2487, NS
IFN-γ	1.916 (±0.161)	3.040 (±0.107)	<0.895	2.288 (±0.134)	<0.907	a, p = 0.0014, ** b, p = 0.0011, ** c, p < 0.0042, ** d, p = 0.1265, NS e, p < 0.0045, ** f, p < 0.0001, **** g, p = 0.0046, ** h, p < 0.0001, **** i, p < 0.0003, *** k, p < 0.0003, ***	1.719 (±0.121)	2.951 (±0.101)	<0.895	2.478 (±0.275)	<1.756	a, p = 0.0043, ** b, p = 0.0002, *** c, p < 0.0030, ** d, p = 0.0449, * f, p < 0.0001, **** g, p = 0.1571, NS h, p < 0.0002, *** i, p < 0.0066, ** k, p < 0.1126, NS
CCL2	9.685 (±0.105)	10.058 (±0.065)	<1.595	11.677 (±0.163)	8.077 (±0.024)	a, p = 0.0010, ** b, p = 0.0236, * c, p < 0.0001 **** d, p < 0.0001, **** e, p < 0.0001, **** f, p < 0.0001 **** g, p < 0.0001, **** h, p < 0.0001, **** i, p < 0.0001 **** j, p < 0.0001 **** k, p < 0.0001, ****	10.693 (±0.092)	11.098 (±0.059)	<1.688	11.276 (±0.376)	9.143 (±0.380)	a, p = 0.0033, ** b, p = 0.0100, ** c, p < 0.0001, **** d, p = 0.1823, NS e, p = 0.0074, ** f, p < 0.0001, **** g, p = 0.6553, NS h, p = 0.0023, ** i, p < 0.0001, **** j, p < 0.0001, **** k, p = 0.0072, **
CCL3	<2.987	3.423 (±0.046)	<2.987	<2.987	<2.987	a, p = 0.0009, *** b, p < 0.0006, *** f, p < 0.0006, *** g, p < 0.0006, *** h, p < 0.0006, ***	<2.987	4.444 (±0.051)	<2.987	<2.987	<2.987	a, p = 0.0009, *** b, p < 0.0001, **** f, p < 0.0001, **** g, p < 0.0001, **** h, p < 0.0001, ****
CCL4	2.126 (±0.041)	2.960 (±0.042)	<1.531	5.356 (±0.277)	1.969 (±0.154)	a, p = 0.0014, ** b, p < 0.0001, **** c, p < 0.0001 **** d, p < 0.0001, **** e, p = 0.3633, NS f, p < 0.0001 **** g, p = 0.0001, *** h, p = 0.0008, *** i, p < 0.0001 **** j, p < 0.0915, NS k, p < 0.0001, ****	2.328 (±0.044)	3.676 (±0.012)	<1.531	4.627 (±0.285)	2.190 (±0.224)	a, p = 0.0015, ** b, p < 0.0001, **** c, p < 0.0001, **** d, p = 0.0002, *** e, p = 0.5696, NS f, p < 0.0001, **** g, p = 0.0156, * h, p = 0.0006, *** i, p < 0.0003, *** j, p < 0.0831, NS k, p = 0.0005, ***
CCL5	3.165 (±0.023)	6.680 (±0.026)	2.468 (±0.059)	7.844 (±0.123)	7.274 (±0.013)	a, p < 0.0001, **** b, p < 0.0001, **** c, p < 0.0001, **** d, p < 0.0001, **** e, p < 0.0001, **** f, p < 0.0001, **** g, p < 0.0001, **** h, p < 0.0001, **** i, p < 0.0001, **** j, p < 0.0001, **** k, p = 0.0037, **	3.930 (±0.043)	7.497 (±0.041)	2.768 (±0.141)	7.757 (±0.251)	8.422 (±0.335)	a, p < 0.0001, **** b, p < 0.0001, **** c, p = 0.0002, *** d, p < 0.0001, **** e, p < 0.0001, **** f, p < 0.0001, **** g, p = 0.3459, NS h, p = 0.0336, * i, p < 0.0001, **** j, p < 0.0001, **** k, p = 0.1631, NS
CCL7	3.618 (±0.077)	3.619 (±0.097)	<1.672	3.406 (±0.186)	2.224 (±0.101)	a, p = 0.0044, ** b, p = 0.9938, NS c, p < 0.0001, **** d, p = 0.3300, NS e, p < 0.0001, **** f, p < 0.0001, **** g, p = 0.3466, NS h, p < 0.0001, **** i, p < 0.0006, *** j, p < 0.0081, ** k, p = 0.0014, **	3.704 (±0.031)	3.805 (±0.131)	<1.672	3.082 (±0.184)	2.550 (±0.315)	a, p = 0.0022, ** b, p = 0.4817, NS c, p < 0.0001, **** d, p = 0.0156, * e, p = 0.0108, * f, p < 0.0001, **** g, p = 0.0185, * h, p = 0.0104, * i, p < 0.0016, ** j, p < 0.0963, NS k, p = 0.1950, NS
CCL11	3.203 (±0.031)	3.242 (±0.038)	<1.580	1.934 (±0.094)	2.048 (±0.128)	a, p = 0.0021, ** b, p = 0.4583, NS c, p < 0.0001, **** d, p < 0.0001, **** e, p = 0.0001, *** f, p < 0.0001 **** g, p < 0.0001, **** h, p = 0.0001, *** i, p < 0.0368, * j, p < 0.0412, * k, p 0.4999, NS	3.248 (±0.045)	3.335 (±0.033)	<1.580	<1.951	2.036 (±0.076)	a, p = 0.0024, ** b, p = 0.1733, NS c, p < 0.0001, **** d, p < 0.0001, **** e, p < 0.0001, **** f, p < 0.0001, **** g, p < 0.0001, **** h, p < 0.0001, **** j, p < 0.0055, ** k, p < 0.4623, NS
CCL22	3.171 (±0.173)	3.633 (±0.050)	3.115 (±0.088)	3.645 (±0.162)	4.148 (±0.083)	a, p = 0.0001, *** b, p = 0.0426, * c, p = 0.7827, NS d, p = 0.0925, NS e, p = 0.0022, ** f, p = 0.0022, ** g, p = 0.9458, NS h, p = 0.0018, ** i, p = 0.0282, * j, p = 0.0001, *** k, p = 0.0326, *	3.343 (±0.093)	3.878 (±0.137)	3.368 (±0.050)	3.397 (±0.164)	5.867 (±0.541)	a, p < 0.0001, **** b, p = 0.0180, * c, p = 0.8220, NS d, p = 0.7862, NS e, p = 0.0037, ** f, p = 0.0130, * g, p = 0.0659, NS h, p = 0.0118, * i, p = 0.8726, NS j, p = 0.0037, ** k, p = 0.0047, **
CXCL1	13.082 (±0.099)	12.140 (±0.050)	2.005 (±0.134)	8.664 (±0.414)	8.598 (±0.036)	a, p < 0.0001, **** b, p = 0.0001, *** c, p < 0.0001, **** d, p < 0.0001, **** e, p < 0.0001, **** f, p < 0.0001, **** g, p = 0.0002, *** h, p < 0.0001, **** i, p < 0.0001, **** j, p < 0.0001, **** k, p = 0.8800, NS	12.953 (±0.097)	13.348 (±0.105)	<1.828	7.858 (±0.333)	10.319 (±0.471)	a, p = 0.0012, ** b, p = 0.0323, * c, p < 0.0001, **** d, p < 0.0001, **** e, p = 0.0015, ** f, p < 0.0001, **** g, p < 0.0001, **** h, p = 0.0008, *** i, p < 0.0001, **** j, p < 0.0001, **** k, p = 0.0053, **
CXCL10	7.159 (±0.053)	8.538 (±0.064)	<0.523	11.396 (±0.370)	6.231 (±0.042)	a, p = 0.0011, ** b, p < 0.0001, **** c, p < 0.0001, **** d, p < 0.0001, **** e, p < 0.0001, **** f, p < 0.0001, **** g, p = 0.0003, *** h, p < 0.0001, **** i, p < 0.0001, **** j, p < 0.0001, **** k, p < 0.0001, ****	7.239 (±0.054)	9.593 (±0.101)	2.316 (±0.124)	11.263 (±0.576)	7.466 (±0.486)	a, p < 0.0001, **** b, p < 0.0001, **** c, p < 0.0001, **** d, p = 0.0004, *** e, p = 0.6597, NS f, p < 0.0001, **** g, p = 0.0290, * h, p = 0.0052, ** i, p < 0.0001, **** j, p < 0.0001, **** k, p = 0.0024, **
CX3CL1	5.609 (±0.118)	7.360 (±0.070)	4.419 (±0.101)	5.476 (±0.078)	6.513 (±0.102)	a, p < 0.0001, **** b, p < 0.0001, **** c, p = 0.0003, *** d, p = 0.0318, NS e, p = 0.0012, ** f, p < 0.0001, **** g, p < 0.0001, **** h, p = 0.0005, *** i, p = 0.0002, *** j, p < 0.0001, **** k, p = 0.0002, ***	5.908 (±0.069)	8.333 (±0.050)	4.354 (±0.061)	5.657 (±0.220)	8.119 (±0.550)	a, p < 0.0001, **** b, p < 0.0001, **** c, p < 0.0001, **** d, p = 0.3188, NS e, p = 0.0072, ** f, p < 0.0001, **** g, p < 0.0001, **** h, p = 0.7118, NS i, p = 0.0012, ** j, p = 0.0005, *** k, p = 0.0060, **
IL-1α	3.165 (±0.048)	4.126 (±0.015)	<1.356	2.393 (±0.061)	<1.356	a, p = 0.0010, ** b, p < 0.0001, **** c, p < 0.0001, **** d, p < 0.0001, **** e, p < 0.0001, **** f, p < 0.0001, **** g, p < 0.0001, **** h, p < 0.0001, **** i, p < 0.0001, **** k, p < 0.0001, ****	3.608 (±0.213)	5.154 (±0.114)	<1.356	2.686 (±0.128)	<1.356	a, p = 0.0010, ** b, p = 0.0007, *** c, p < 0.0003, *** d, p = 0.0100, * e, p < 0.0003, *** f, p < 0.0001, **** g, p < 0.0001, **** h, p < 0.0001, **** i, p < 0.0003, *** k, p < 0.0003, ***
IL-1β	<1.470	<1.470	<1.470	<1.470	<1.470		<1.470	<1.470	<1.470	<1.470	<1.470	
IL-2	<1.417	<1.417	<1.417	<1.417	<1.417		<1.417	<1.417	<1.417	<1.417	<1.417	
IL-3	<1.362	<1.362	<1.362	<1.362	<1.362		<1.362	<1.362	<1.362	<1.362	<1.362	
IL-4	6.209 (±0.121)	5.929 (±0.131)	<1.417	3.788 (±0.199)	3.029 (±0.085)	a, p = 0.0013, ** b, p = 0.1676, NS c, p < 0.0001, **** d, p < 0.0001, **** e, p < 0.0001, **** f, p < 0.0001, **** g, p = 0.0001, *** h, p < 0.0001, **** i, p < 0.0002, *** j, p < 0.0001, **** k, p = 0.0126, *	6.253 (±0.123)	6.448 (±0.080)	<1.417	3.142 (±0.306)	4.301 (±0.490)	a, p = 0.0018, ** b, p = 0.2335, NS c, p < 0.0001, **** d, p < 0.0001, **** e, p = 0.0083, ** f, p < 0.0001, **** g, p < 0.0001, **** h, p = 0.0050, ** i, p < 0.0072, ** j, p < 0.0059, ** k, p = 0.0915, NS
IL-5	<1.480	<1.480	<1.480	<1.480	<1.480		<1.480	<1.480	<1.480	<1.480	<1.480	
IL-6	4.409 (±0.058)	12.161 (±0.038)	<1.189	<1.189	1.932 (±0.096)	a, p = 0.0010, ** b, p < 0.0001, **** c, p < 0.0001, **** d, p < 0.0001, **** e, p < 0.0001, **** f, p < 0.0001, **** g, p < 0.0001, **** h, p < 0.0001, **** j, p < 0.0015, ** k, p < 0.0015, **	5.532 (±0.135)	12.378 (±0.132)	<1.189	<1.300	2.141 (±0.064)	a, p = 0.0011, ** b, p < 0.0001, **** c, p < 0.0001, **** d, p < 0.0001, **** e, p < 0.0001, **** f, p < 0.0001, **** g, p < 0.0001, **** h, p < 0.0001, **** j, p < 0.0001, **** k, p < 0.0001, ****
IL-7	2.214 (±0.155)	4.023 (±0.109)	1.260 (±0.432)	2.474 (±0.302)	2.717 (±0.401)	a, p = 0.0003, *** b, p < 0.0001, **** c, p = 0.0831, NS d, p = 0.4721, NS e, p = 0.2865, NS f, p = 0.0008, *** g, p = 0.0029, ** h, p = 0.0200, * i, p = 0.0610, NS j, p = 0.0485, * k, p = 0.6462, NS	2.717 (±0.178)	4.087 (±0.121)	<0.803	2.619 (±0.344)	2.644 (±0.271)	a, p = 0.0054, ** b, p = 0.0007, *** c, p < 0.0003, *** d, p = 0.8088, NS e, p = 0.8299, NS f, p < 0.0001, **** g, p = 0.0069, ** h, p = 0.0028, ** i p < 0.0098, ** j, p < 0.0030, ** k, p = 0.9559, NS
IL-8	12.551 (±0.103)	12.427 (±0.124)	<0.807	4.481 (±0.350)	8.117 (±0.057)	a, p = 0.0015, ** b, p = 0.4705, NS c, p < 0.0001, **** d, p < 0.0001, **** e, p < 0.0001, **** f, p < 0.0001, **** g, p < 0.0001, **** h, p < 0.0001, **** i, p < 0.0003, *** j, p < 0.0001, **** k, p < 0.0001, ****	>12.785	>12.970	<0.807	4.925 (±0.406)	8.715 (±0.334)	a, p = 0.0011, ** c, p < 0.0001, **** d, p < 0.0001, **** e, p < 0.0001, **** f, p < 0.0001, **** g, p < 0.0001, **** h, p < 0.0001, **** i, p < 0.0004, *** j, p < 0.0001, **** k, p = 0.0004, ***
IL-9	<1.575	<1.575	<1.575	<1.838	<1.575	a, p = 0.0099 **	<1.575	<1.575	<1.575	2.334 (±0.178)	<1.575	a, p = 0.0009, *** d, p < 0.0236, NS g, p < 0.0236, NS i, p < 0.0236, NS k, p < 0.0236, NS
IL-10	1.421 (±0.125)	3.234 (±0.118)	<0.111	1.357 (±0.209)	0.504	a, p = 0.0021, ** b, p < 0.0001, **** c, p < 0.0003, *** d, p = 0.8024, NS e, p < 0.0021, ** f, p < 0.0001, **** g, p = 0.0002, *** h, p < 0.0001, **** i, p < 0.0056, **** k, p < 0.0279, *	1.569 (±0.135)	3.583 (±0.149)	<0.111	1.461 (±0.195)	<1.002	a, p = 0.0039, ** b, p < 0.0001, **** c, p < 0.0003, *** d, p = 0.6646, NS e, p < 0.0249, NS f, p < 0.0001, **** g, p = 0.0001, *** h, p < 0.0001, *** i, p < 0.0027, ** k, p < 0.1474
IL-12p40	2.248 (±0.094)	2.707 (±0.110)	<1.251	2.748 (±0.121)	1.747 (±0.115)	a, p < 0.0001, **** b, p = 0.0194, * c, p < 0.0003, *** d, p = 0.0173, * e, p = 0.0148, * f, p < 0.0001, **** g, p = 0.8103, NS h, p = 0.0009, *** i, p < 0.0001, **** j, p < 0.0224, * k, p = 0.0010, ***	2.464 (±0.076)	2.962 (±0.048)	<0.900	2.535 (±0.141)	2.047 (±0.133)	a, p = 0.0025, ** b, p = 0.0014, ** c, p < 0.0001, **** d, p = 0.6744, NS e, p = 0.0346, * f, p < 0.0001, **** g, p 0.0288, * h, p = 0.0006, *** i, p < 0.0002, *** j, p < 0.0009, *** k, p = 0.4459, *
IL-12p70	<0.973	1.675 (±0.121)	<0.350	<0.613	<0.401	a, p = 0.0198, * b, p < 0.0064, ** c, p = 0.0160, * d, p = 0.3972, NS e, p = 0.0145, * f, p < 0.0002, *** g, p < 0.0008, *** h, p < 0.0003, *** i, p = 0.0550, NS j, p = 0.4226, NS k, p = 0.0322, *	<1.086	1.987 (±0.243)	<0.241	<0.928	<0.758	a, p = 0.0425, * b, p < 0.0395, * f, p < 0.0023, ** g, p < 0.0216, * h, p < 0.0117, *
IL-13	1.694 (±0.086)	2.566 (±0.074)	<0.956	2.658 (±0.054)	<1.140	a, p = 0.0017, ** b, p = 0.0003, *** c, p < 0.0009, *** d, p < 0.0001, **** e, p < 0.0039, ** f, p < 0.0001, **** g, p = 0.3510, NS h, p < 0.0001, **** i, p < 0.0001, **** k, p < 0.0001, ****	2.487 (±0.071)	2.994 (±0.039)	<0.956	2.516 (±0.205)	<1.773	a, p = 0.0034, ** b, p = 0.0008, *** c, p < 0.0001, **** d, p = 0.8974, NS e, p < 0.0004, *** f, p < 0.0001, **** g, p = 0.0625, NS h, p < 0.0001, **** i, p < 0.0017, ** k, p < 0.0430, *
IL-15	2.026 (±0.102)	3.995 (±0.025)	< −0.286	2.083 (±0.288)	0.882 (±0.042)	a, p = 0.0015, ** b, p < 0.0001, **** c, p < 0.0001, **** d, p = 0.8593, NS e, p < 0.0001, **** f, p < 0.0001, **** g, p = 0.0006, *** h, p < 0.0001, **** i, p < 0.0011, ** j, p < 0.0001, **** k, p = 0.0062, **	2.755 (±0.041)	6.648 (±0.026)	< −0.148	1.887 (±0.199)	1.776 (±0.154)	a, p = 0.0016, ** b, p < 0.0001, **** c, p < 0.0001, **** d, p = 0.0053, ** e, p = 0.0009, *** f, p < 0.0001, **** g, p < 0.0001, **** h, p < 0.0001, **** i, p < 0.0004, *** j, p < 0.0001, **** k, p = 0.6748, NS
IL-17A	0.954 (±0.136)	1.405 (±0.105)	<0.422	<0.783	<0.500	a, p = 0.0053, ** b, p = 0.0390, * c, p < 0.0327, * d, p < 0.4101, NS e, p < 0.0001, **** f, p < 0.0006, *** g, p < 0.0057, ** h, p < 0.0001, **** k, p = 0.0809, NS	1.075 (±0.126)	1.604 (±0.216)	<0.422	<0.819	<0.693	a, p = 0.0074, ** b, p = 0.0786, NS c, p < 0.0105, * d, p < 0.2005, NS e, p < 0.0755, NS f, p < 0.0082, ** g, p < 0.0422, * h, p < 0.0244, *
IL-1RA	3.149 (±0.082)	4.175 (±0.077)	2.033 (±0.154)	3.231 (±0.195)	2.474 (±0.095)	a, p < 0.0001, **** b, p < 0.0001, **** c, p = 0.0007, *** d, p = 0.7134, NS e, p = 0.0017, ** f, p < 0.0001, **** g, p = 0.0041, ** h, p < 0.0001, **** i, p = 0.0030, ** j, p = 0.0506, NS k, p = 0.0130, *	3.710 (±0.105)	4.600 (±0.099)	2.021 (±0.108)	3.180 (±0.200)	3.661 (±0.426)	a, p < 0.0001, **** b, p = 0.0008, *** c, p < 0.0001, **** d, p = 0.0572, NS e, p = 0.9164, NS f, p < 0.0001, **** g, p = 0.0007, *** h, p = 0.0755, NS i, p = 0.0022, ** j, p = 0.0097, ** k, p = 0.3451, NS
G-CSF	8.465 (±0.125)	8.400 (±0.026)	2.145 (±0.067)	5.703 (±0.113)	4.874 (±0.267)	a, p < 0.0001, **** b, p = 0.6295, NS c, p < 0.0001, **** d, p < 0.0001, **** e, p < 0.0001, **** f, p < 0.0001, **** g, p < 0.0001, **** h, p < 0.0001, **** i, p < 0.0001, **** j, p < 0.0001, **** k, p = 0.0288, *	8.720 (±0.158)	10.482 (±0.181)	2.489 (±0.076)	6.173 (±0.193)	5.032 (±0.301)	a, p < 0.0001, **** b, p = 0.0003, *** c, p < 0.0001, **** d, p < 0.0001, **** e, p < 0.0001, **** f, p < 0.0001, **** g, p < 0.0001, **** h, p < 0.0001, **** i, p < 0.0001, **** j, p = 0.0002, *** k, p = 0.0188, *
GM-CSF	4.872 (±0.075)	10.063 (±0.023)	<1.546	2.972 (±0.146)	<1.573	a, p = 0.0011, ** b, p < 0.0001, **** c, p < 0.0001, **** d, p < 0.0001, **** e, p = 0.0003, *** f, p < 0.0001, **** g, p < 0.0001, **** h, p < 0.0001, **** i, p < 0.0005, *** k, p = 0.0273, *	5.203 (±0.150)	11.917 (±0.038)	<1.546	2.957 (±0.223)	<2.059	a, p = 0.0011, ** b, p < 0.0001, **** c, p < 0.0001, **** d, p = 0.0002, *** e, p < 0.0001, **** f, p < 0.0001, **** g, p < 0.0001, **** h, p < 0.0001, **** i, p < 0.0043, ** k, p < 0.0296, *
FLT3L	3.027 (±0.056)	4.010 (±0.026)	<1.525	3.439 (±0.197)	3.063 (±0.050)	a, p = 0.0037, ** b, p < 0.0001, **** c, p < 0.0001, **** d, p = 0.0911, NS e, p = 0.6482, NS f, p < 0.0001, **** g, p = 0.0282, * h, p < 0.0001, **** i, p < 0.0005, *** j, p < 0.0001, **** k, p = 0.1138, NS	3.316 (±0.046)	4.587 (±0.016)	<1.459	2.941 (±0.211)	4.199 (±0.365)	a, p = 0.0039, ** b, p < 0.0001, **** c, p < 0.0001, **** d, p = 0.1332, NS e, p = 0.0533, NS f, p < 0.0001, **** g, p = 0.0002, *** h, p = 0.3288, NS i, p < 0.0025, ** j, p < 0.0018, ** k, p = 0.0245, *
sCD40L	1.745 (±0.252)	2.467 (±0.199)	<1.007	2.093 (±0.227)	<1.053	a, p = 0.0041, ** b, p = 0.0651, NS c, p < 0.0835, NS d, p = 0.3442, NS e, p < 0.0997, NS f, p < 0.0020, ** g, p = 0.2602, NS h, p < 0.0024, ** i, p < 0.0148, * k, p < 0.0177, *	2.061 (±0.134)	2.876 (±0.147)	<1.007	2.034 (±0.347)	<1.603	a, p = 0.0071, ** b, p = 0.0063, ** c, p < 0.0014, ** d, p = 0.9445, NS e, p < 0.0001, **** f, p < 0.0001, **** g, p = 0.0668, NS h, p < 0.0001, **** i, p < 0.0816, NS k, p < 0.0031, **
EGF	2.505 (±0.045)	2.771 (±0.065)	1.938 (±0.087)	2.207 (±0.080)	2.256 (±0.176)	a, p = 0.0004, *** b, p = 0.0147, * c, p = 0.0012, ** d, p = 0.0174, * e, p = 0.2187, NS f, p = 0.0003, *** g, p = 0.0016, ** h, p = 0.0333, * i, p = 0.0640, NS j, p = 0.1559, NS k, p = 0.8062, NS	2.596 (±0.039)	2.820 (±0.084)	1.895 (±0.051)	2.311 (±0.142)	<1.971	a, p = 0.0065, ** b, p = 0.0516, NS c, p < 0.0001, **** d, p = 0.1014, NS e, p = 0.0747, NS f, p < 0.0001, **** g, p = 0.0215, * h, p = 0.0286, * i, p = 0.0329, * j, p = 0.3603, NS k, p = 0.4962, NS
FGF-2	4.936 (±0.144)	5.522 (±0.106)	3.197 (±0.183)	4.806 (±0.208)	3.990 (±0.093)	a, p < 0.0001, **** b, p = 0.0168, * c, p = 0.0003, *** d, p = 0.6269, NS e, p = 0.0015, ** f, p < 0.0001, **** g, p = 0.0222, * h, p < 0.0001, **** i, p = 0.0012, ** j, p = 0.0083, ** k, p = 0.0117, *	7.333 (±0.032)	5.404 (±0.108)	3.365 (±0.246)	4.627 (±0.218)	4.599 (±0.277)	a, p < 0.0001, **** b, p < 0.0001, **** c, p < 0.0001, **** d, p < 0.0001, **** e, p < 0.0001, **** f, p = 0.0003, *** g, p = 0.0189, * h, p = 0.0355, * i, p = 0.0086, ** j, p = 0.0159, * k, p = 0.9399, NS
TGFα	<1.485	3.345 (±0.030)	3.223 (±0.038)	3.124 (±0.186)	<1.485	a, p = 0.0038, ** b, p < 0.0001, **** c, p < 0.0001, **** d, p < 0.0008, *** f, p = 0.0459, * g, p = 0.2839, NS h, p < 0.0001, **** i, p = 0.6189, NS j, p < 0.0001, **** k, p < 0.0008, ***	<1.485	3.727 (±0.042)	3.836 (±0.025)	2.571 (±0.148)	1.895 (±0.172)	a, p = 0.0013, ** b, p < 0.0001, **** c, p < 0.0001, **** d, p < 0.0020, **** e, p < 0.1425 f, p = 0.0662, NS g, p = 0.0003, *** h, p < 0.0001, **** i, p = 0.0001, *** j, p < 0.0001, **** k, p = 0.0242, *
PDGF-AA	5.778 (±0.056)	6.424 (±0.029)	5.393 (±0.030)	5.740 (±0.447)	5.507 (±0.020)	a, p = 0.0228, * b, p < 0.0001, **** c, p = 0.0009, *** d, p = 0.9343, NS e, p = 0.0038, ** f, p < 0.0001, **** g, p = 0.1772, NS h, p < 0.0001, **** i, p = 0.4781, NS j, p = 0.0198, * k, p = 0.6212, NS	6.627 (±0.051)	7.486 (±0.011)	6.700 (±0.046)	5.256 (±0.408)	9.247 (±0.394)	a, p < 0.0001, **** b, p < 0.0001, **** c, p = 0.3251, NS d, p = 0.0158, * e, p = 0.0006, *** f, p < 0.0001, **** g, p = 0.0016, ** h, p = 0.0042, ** i, p = 0.0126, * j, p = 0.0007, *** k, p = 0.0004, ***
PDGF-AB/BB	3.588 (±0.104)	5.179 (±0.033)	7.647 (±0.095)	8.473 (±0.486)	4.841 (±0.048)	a, p < 0.0001, **** b, p < 0.0001, **** c, p < 0.0001, **** d, p < 0.0001, **** e, p < 0.0001, **** f, p < 0.0001, **** g, p = 0.0005, *** h, p = 0.0012, ** i, p = 0.1461, NS j, p < 0.0001, **** k, p = 0.0003, ***	3.598 (±0.101)	6.221 (±0.020)	9.271 (±0.015)	8.043 (±0.190)	10.309 (±0.176)	a, p < 0.0001, **** b, p < 0.0001, **** c, p < 0.0001, **** d, p < 0.0001, **** e, p < 0.0001, **** f, p < 0.0001, **** g, p < 0.0001, **** h, p < 0.0001, **** i, p = 0.0007, *** j, p = 0.0011, ** k, p = 0.0001, ***
VEGF-A	7.239 (±0.053)	10.785 (±0.093)	6.619 (±0.047)	7.846 (±0.457)	7.757 (±0.024)	a, p < 0.0001, **** b, p < 0.0001, **** c, p = 0.0001, *** d, p = 0.2352, NS e, p = 0.0001, *** f, p < 0.0001, **** g, p = 0.0007, *** h, p < 0.0001, **** i, p = 0.0371, * j, p < 0.0001, **** k, p = 0.8519, NS	8.110 (±0.051)	11.014 (±0.036)	7.774 (±0.034)	7.846 (±0.345)	8.304 (±0.039)	a, p < 0.0001, **** b, p < 0.0001, **** c, p = 0.0015, ** d, p = 0.4779, NS e, p = 0.0229, * f, p < 0.0001, **** g, p < 0.0001, **** h, p < 0.0001, **** i, p = 0.8417, NS j, p < 0.0001, **** k, p = 0.2347, NS

Cytokine levels are presented in log scale, base 2 (±SEM). Each value is the mean of measurements from 4 independent setups. In the case of values below or above detection level, means and p-values are reported using “less than” or “greater than” symbols. a, ANOVA; b, MCF-10A *vs.* MDA-MB-231; c, MCF-10A *vs.* MCF-7; d, MCF-10A *vs.* T-47D; e, MCF-10A *vs.* BT-474; f, MDA-MB-231 *vs.* MCF-7; g, MDA-MB-231 *vs.* T-47D; h, MDA-MB-231 *vs.* BT-474; i, MCF-7 *vs.* T-47D; j, MCF-7 *vs.* BT-474; k, T-47D *vs.* BT-474; NS, non-significant (p > 0.05); *p ≤ 0.05; **p ≤ 0.01; ***p ≤ 0.001; ****p ≤ 0.0001.

## 2 Experimental design, materials and methods

### 2.1 Cell lines

MDA-MB-231 (Cat. # HTB-26) and MCF-7 (Cat. # HTB-22) cells were purchased from the American Type Culture Collection (ATCC). T-47D, BT-474, and MCF-10A cells were a generous gift from Dr. Dipali Sharma's laboratory at Sidney Kimmel Comprehensive Cancer Center at the Johns Hopkins University in Baltimore, Maryland. MDA-MB-231, MCF-7, T-47D, and BT-474 cells were grown in Dulbecco's Modified Eagle's Medium (DMEM) (Cat. # 10-013-CF, Corning) supplemented with 10% fetal bovine serum (FBS) (Cat. # 1500–500, VWR International) and antibiotic/antimycotic solution (containing 10,000 IU penicillin, 10,000 μg/ml streptomycin, and 25 μg/ml amphotericin B) (Cat. # 30-004-CI, Corning, Inc.) at 37 °C/5% CO_2_. MCF-10A cells were grown in Gibco HuMEC Ready Medium kit (Thermo Fisher Scientific) containing HuMEC Basal Medium, HuMEC supplement, and bovine pituitary extract (BPE) additionally supplemented with 10% FBS and antibiotic/antimycotic mixture.

### 2.2 Experimental conditions

For experiments, 0.3 × 10^6^ cells were seeded in 6-well plates containing complete growing cell culture medium. On the following day, the medium in each well was replaced with 1 ml serum-free medium, and the cells were incubated for an additional 24 or 48 hours. After the experiment, the cell culture conditioned medium was collected and centrifuged to remove all dead cells and debris, and stored at −86 °C until further analyzed.

### 2.3 LLIPLEX assay

Cytokine levels in cell culture conditioned medium were measured using 41 cytokine MILLIPLEX assay kit [Cat. # HCYTMAG-60K-PX41, MilliporeSigma] and MAGPIX Multiplexing System [MilliporeSigma] following the manufacturer's protocol. Data were analyzed using xPONENT4.2 and Milliplex Analyst 5.1 data analysis software [MilliporeSigma].

### 2.4 Statistical analyses

Data from four independent experiment setups were presented as mean ± SEM and analyzed on the logarithmic scale base 2. One-way ANOVA tests (if complete data) or Kruskal-Wallis tests (if some out-of-range data) were used to assess overall significance. Independent t-tests were used for post-hoc pairwise comparisons. T-tests with summary data were used if one of the two groups had values below or above detection level. For overall tests, data were considered statistically significant at the p < 0.05 level of significance. For post-hoc tests, a Bonferoni-adjusted significance level of p < 0.005 is recommended. All analyses were performed using SAS version 9.4 (SAS Institute).
